# Supplementary material for: Navigating agricultural nonpoint source pollution governance: A social network analysis of best management practices in central Pennsylvania
Source: PLoS One. 2024 May 23;19(5):e0303745. doi: 10.1371/journal.pone.0303745 (PMC11115221; doi:10.1371/journal.pone.0303745)
Supplement: S1 File — I. questionnaire for farmers; II. Questionnaire for organizations. (DOCX) [file pone.0303745.s001.docx]

**S1 File**

**Survey questionnaires**. I. questionnaire for farmers ; II. Questionnaire for organizations.

1. **QUESTIONNAIRE FOR FARMERS INTERVIEW**

# **Section A. General information and farm typology**

# Gender:

# Age:

# Labor force Number of Annual Work Units (AWU): Proportion of family labor (% of AWU):

# Area farmed: hectares / acres (please circle)

# Number of animals: heads / Animal Units Equivalent (AUE) (please circle)

# Stocking rate (AUE/ha or AUE/ac), if applicable:

# **Section B. Actors and messages data**

This section intends to collect information about (1) the actors in your network with whom you share (send/receive) information in relation to practices on your farm, and (2) to collect and characterize the information fluxes that influence the practices that you apply on your farm.

Please fill up the table below by indicating:

- In column A: which of the practices listed you apply on your farm **(Yes/No)**.
- In Column B1 and C1: the actor(s) from whom you receive messages relating to this practice (B1), and the actors to whom you send messages relating to this practice (C1). **Consider all sources of information and knowledge** – can include informal network - such as other farmers, family, social media, industry representatives, advisory etc. Also consider formal contacts – such as the department of agriculture.
- In column B2 and C2: the type of message received or emitted. **Consider all types of information** (news, knowledge, money, advice, etc.)
- In column B3 and C3: the frequency at which you receive or emit the message: Daily **(D)**, Weekly **(W)**, Monthly **(M)**, every 2 months **(S)**, Bi-Annually **(B)**, Annually or less than annually **(A)**.
- In column B4: your action, how responded/reacted to this message: High potential-will apply **(H)**, Medium-high potential-should do **(MH)**, Medium potential-may do or will reflect on it **(M)**, Low potential-unlikely **(L)**.

| **Do you apply any of these practices on your farm?** | **Column A:**  yes **(Y)**,  no **(N)**,  *not applicable (n/a)* | **Column B1:**  actor(s) from whom you receive related messages | **Column B2:** *knowledge, money, advice, etc.* | **Column B3:** frequency  **(W, M, S, B, A)** | **Column B4:** action / response **(H, MH, M, L)** | **Column C1:** actor(s) to whom you send related information/  messages | **Column C2:** *knowledge, money, advice, etc.* | **Column C3:** frequency **(W, M, S, B, A)** |
| --- | --- | --- | --- | --- | --- | --- | --- | --- |
| Forested riparian buffers |  |  |  |  |  |  |  |  |
|  |  |  |  |  |  |  |  |  |
|  |  |  |  |  |  |  |  |  |
|  |  |  |  |  |  |  |  |  |
|  |  |  |  |  |  |  |  |  |
| Grass riparian buffers |  |  |  |  |  |  |  |  |
|  |  |  |  |  |  |  |  |  |
|  |  |  |  |  |  |  |  |  |
|  |  |  |  |  |  |  |  |  |
|  |  |  |  |  |  |  |  |  |
| Cover-crops (unharvested) |  |  |  |  |  |  |  |  |
|  |  |  |  |  |  |  |  |  |
|  |  |  |  |  |  |  |  |  |
|  |  |  |  |  |  |  |  |  |
|  |  |  |  |  |  |  |  |  |
| No-till or conservation tillage |  |  |  |  |  |  |  |  |
|  |  |  |  |  |  |  |  |  |
|  |  |  |  |  |  |  |  |  |
|  |  |  |  |  |  |  |  |  |
|  |  |  |  |  |  |  |  |  |
| Prescribed grazing |  |  |  |  |  |  |  |  |
|  |  |  |  |  |  |  |  |  |
|  |  |  |  |  |  |  |  |  |
|  |  |  |  |  |  |  |  |  |
|  |  |  |  |  |  |  |  |  |
| Permanent grasslands or wetlands maintenance |  |  |  |  |  |  |  |  |
|  |  |  |  |  |  |  |  |  |
|  |  |  |  |  |  |  |  |  |
|  |  |  |  |  |  |  |  |  |
|  |  |  |  |  |  |  |  |  |
| Contour grass strips or strip cropping |  |  |  |  |  |  |  |  |
|  |  |  |  |  |  |  |  |  |
|  |  |  |  |  |  |  |  |  |
|  |  |  |  |  |  |  |  |  |
|  |  |  |  |  |  |  |  |  |
| Hedge rows plantation / maintenance |  |  |  |  |  |  |  |  |
|  |  |  |  |  |  |  |  |  |
|  |  |  |  |  |  |  |  |  |
|  |  |  |  |  |  |  |  |  |
|  |  |  |  |  |  |  |  |  |
| Precision feeding |  |  |  |  |  |  |  |  |
|  |  |  |  |  |  |  |  |  |
|  |  |  |  |  |  |  |  |  |
|  |  |  |  |  |  |  |  |  |
|  |  |  |  |  |  |  |  |  |
| Reduced stocking rates (< 1.5 AUE / ha, or < 3.7 AUE / ac) |  |  |  |  |  |  |  |  |
|  |  |  |  |  |  |  |  |  |
|  |  |  |  |  |  |  |  |  |
|  |  |  |  |  |  |  |  |  |
|  |  |  |  |  |  |  |  |  |
| Animal exclusion (fencing from rivers, and streams) |  |  |  |  |  |  |  |  |
|  |  |  |  |  |  |  |  |  |
|  |  |  |  |  |  |  |  |  |
|  |  |  |  |  |  |  |  |  |
|  |  |  |  |  |  |  |  |  |
| Manure storage facility (sufficient capacity, water tight, above water table, etc.) |  |  |  |  |  |  |  |  |
|  |  |  |  |  |  |  |  |  |
|  |  |  |  |  |  |  |  |  |
|  |  |  |  |  |  |  |  |  |
|  |  |  |  |  |  |  |  |  |
| Having a manure management plan (written down) |  |  |  |  |  |  |  |  |
|  |  |  |  |  |  |  |  |  |
|  |  |  |  |  |  |  |  |  |
|  |  |  |  |  |  |  |  |  |
|  |  |  |  |  |  |  |  |  |
| Following a manure management plan |  |  |  |  |  |  |  |  |
|  |  |  |  |  |  |  |  |  |
|  |  |  |  |  |  |  |  |  |
|  |  |  |  |  |  |  |  |  |
|  |  |  |  |  |  |  |  |  |
| Following a fertilization management plan (NPK other than manure) |  |  |  |  |  |  |  |  |
|  |  |  |  |  |  |  |  |  |
|  |  |  |  |  |  |  |  |  |
|  |  |  |  |  |  |  |  |  |
|  |  |  |  |  |  |  |  |  |
| Precision fertilization technology (e.g. manure injection) |  |  |  |  |  |  |  |  |
|  |  |  |  |  |  |  |  |  |
|  |  |  |  |  |  |  |  |  |
|  |  |  |  |  |  |  |  |  |
|  |  |  |  |  |  |  |  |  |
| Other practice contributing to water purification /regulation? |  |  |  |  |  |  |  |  |
|  |  |  |  |  |  |  |  |  |
|  |  |  |  |  |  |  |  |  |
|  |  |  |  |  |  |  |  |  |
|  |  |  |  |  |  |  |  |  |

1. **QUESTIONNAIRE FOR ORGANIZATIONS INTERVIEW**

**Section A. General information**

- Organization / structure name (optional):
- Organization / structure purpose:
- Please indicate your role in the organization (optional):
- Please indicate the organizations scale and scope of action (please circle):

Local (municipal, township, county), State, Chesapeake Bay Watershed, Federal, International

- Please check the boxes that apply to you organization / structure (please check those that apply if more than one)

| Agricultural inputs supplier (equipment, fertilizers, feed, seeds, veterinary) |  | Private consultancy - advisory |  | Farmers’ cooperative / farmer’s led organization |  |
| --- | --- | --- | --- | --- | --- |
| Agricultural service provider (business, except consultancy-advisory) |  | Public advisory |  | NGO |  |
| Processor / manufacturer |  | Research and development |  | Lobby |  |
| Wholesaler |  | Regulatory and control agency (government) |  | Civil society, public, consumer |  |
| Retailer |  | Private funding agency |  | Civil society, farmers private sphere |  |
| Certifier / accredited certification body |  | Public funding agency |  | Other (please specify) |  |

**Section B. Actors and information fluxes**

This section intends to collect information about (1) the actors in your network with whom you share (send/receive) information in relation to best management practices, and (2) the type and frequency of occurrence of the information fluxes.

Please fill up the table below by indicating:

- In Column B1 and C1: the actor(s) from whom you receive information relating to this practice (B1), and the actors to whom you send information relating to this practice (C1). **Please consider all sources of information and knowledge** – can include informal network - such as other farmers, family, social media, industry representatives, advisory etc. Also consider formal contacts – such as the department of agriculture.
- In column B2 and C2: the type of information received or emitted **(refer to numbering)**. **Please consider all types of information**, including provision of the service or request for the service: Relating to personalized technical assistance **(1)**; Relating to general knowledge **(2);** relating to funding **(3)**; relating to regulations-standards-programs **(4)**; networking-request to get connected or connecting someone with another organization **(5)**.
- In column B3 and C3: the frequency at which you receive or emit the information **(refer to letter codes)**: Daily More than once a month **(W)**, Every month to every two months **(M)**, Quarterly to bi-annually **(Q)**, Annually or less than annually **(A)**.

| **Farm practice** | **Column B1:**  actor(s) from whom you receive related information | **Column B2:**  Type  **(1, 2, 3, 4, 5)** | **Column B3:** frequency  **(W, M, Q, A)** | **Column C1:**  actor(s) to whom you send related information | **Column C2:**  Type  **(1, 2, 3, 4, 5)** | **Column C3:** frequency  **(W, M, Q, A)** |
| --- | --- | --- | --- | --- | --- | --- |
| Forested riparian buffers |  |  |  |  |  |  |
|  |  |  |  |  |  |  |
|  |  |  |  |  |  |  |
|  |  |  |  |  |  |  |
|  |  |  |  |  |  |  |
| Grass riparian buffers |  |  |  |  |  |  |
|  |  |  |  |  |  |  |
|  |  |  |  |  |  |  |
|  |  |  |  |  |  |  |
|  |  |  |  |  |  |  |
| Cover-crops (unharvested) |  |  |  |  |  |  |
|  |  |  |  |  |  |  |
|  |  |  |  |  |  |  |
|  |  |  |  |  |  |  |
|  |  |  |  |  |  |  |
| No-till or conservation tillage |  |  |  |  |  |  |
|  |  |  |  |  |  |  |
|  |  |  |  |  |  |  |
|  |  |  |  |  |  |  |
|  |  |  |  |  |  |  |
| Prescribed grazing |  |  |  |  |  |  |
|  |  |  |  |  |  |  |
|  |  |  |  |  |  |  |
|  |  |  |  |  |  |  |
|  |  |  |  |  |  |  |
| Permanent grasslands or wetlands maintenance |  |  |  |  |  |  |
|  |  |  |  |  |  |  |
|  |  |  |  |  |  |  |
|  |  |  |  |  |  |  |
|  |  |  |  |  |  |  |
| Contour grass strips or strip cropping |  |  |  |  |  |  |
|  |  |  |  |  |  |  |
|  |  |  |  |  |  |  |
|  |  |  |  |  |  |  |
|  |  |  |  |  |  |  |
| Hedge rows plantation / maintenance |  |  |  |  |  |  |
|  |  |  |  |  |  |  |
|  |  |  |  |  |  |  |
|  |  |  |  |  |  |  |
|  |  |  |  |  |  |  |
| Precision feeding |  |  |  |  |  |  |
|  |  |  |  |  |  |  |
|  |  |  |  |  |  |  |
|  |  |  |  |  |  |  |
|  |  |  |  |  |  |  |
| Reduced stocking density (< 1.5 AUE / ha, or < 3.7 AUE / ac) |  |  |  |  |  |  |
|  |  |  |  |  |  |  |
|  |  |  |  |  |  |  |
|  |  |  |  |  |  |  |
|  |  |  |  |  |  |  |
| Animal exclusion (fencing from rivers, and streams) |  |  |  |  |  |  |
|  |  |  |  |  |  |  |
|  |  |  |  |  |  |  |
|  |  |  |  |  |  |  |
|  |  |  |  |  |  |  |
| Manure storage facility (capacity, water tight, above water table, etc.) |  |  |  |  |  |  |
|  |  |  |  |  |  |  |
|  |  |  |  |  |  |  |
|  |  |  |  |  |  |  |
|  |  |  |  |  |  |  |
| Having a manure management plan (written down) |  |  |  |  |  |  |
|  |  |  |  |  |  |  |
|  |  |  |  |  |  |  |
|  |  |  |  |  |  |  |
|  |  |  |  |  |  |  |
| Following a manure management plan |  |  |  |  |  |  |
|  |  |  |  |  |  |  |
|  |  |  |  |  |  |  |
|  |  |  |  |  |  |  |
|  |  |  |  |  |  |  |
| Following a nutrients management plan |  |  |  |  |  |  |
|  |  |  |  |  |  |  |
|  |  |  |  |  |  |  |
|  |  |  |  |  |  |  |
|  |  |  |  |  |  |  |
| Precision fertilization technology (e.g. manure injection) |  |  |  |  |  |  |
|  |  |  |  |  |  |  |
|  |  |  |  |  |  |  |
|  |  |  |  |  |  |  |
|  |  |  |  |  |  |  |
| Other practice contributing to water purification /regulation? |  |  |  |  |  |  |
|  |  |  |  |  |  |  |
|  |  |  |  |  |  |  |
|  |  |  |  |  |  |  |
|  |  |  |  |  |  |  |
