# Supplementary material for: Navigating agricultural nonpoint source pollution governance: A social network analysis of best management practices in central Pennsylvania
Source: PLoS One. 2024 May 23;19(5):e0303745. doi: 10.1371/journal.pone.0303745 (PMC11115221; doi:10.1371/journal.pone.0303745)
Supplement: S2 File — (DOCX) [file pone.0303745.s002.docx]

**S3 File**

**Categorization of message types.**

**(relating to) Technical assistance:**

provision of individual personalized advice and technical support, request for technical assistance.

**(relating to) Knowledge:**

channeling of news and information about events, generic and technical information dissemination through publications, newsletters and press releases, workshops, conferences, seminars, etc.), request for generic or technical information.

**(relating to) Funding:**

monetary fluxes such as provision of grants and loans, financial incentives, requests for financial support, etc.

**(relating to) Regulation and standards:**

compliance control, dissemination of information relating to legal frameworks, government programs, regulations and standards, policy measures, inquiries about regulation, standards and government programs.

**(relating to) Networking:**

connecting actors, referring to someone or to an organization, requesting to be linked to an actor.
