# Supplementary material for: Navigating agricultural nonpoint source pollution governance: A social network analysis of best management practices in central Pennsylvania
Source: PLoS One. 2024 May 23;19(5):e0303745. doi: 10.1371/journal.pone.0303745 (PMC11115221; doi:10.1371/journal.pone.0303745)
Supplement: S3 File — (DOCX) [file pone.0303745.s003.docx]

**S5 File**

**Guiding questions for semi-structured interviews.**

*(for both farmers and non-farmers interviewees)*

- What do you think needs to be done more to enhance water quality in the region?
- In your opinion, what critically needs to be addressed to reduce nonpoint source pollution from agricultural land? (not technically, but from a governance perspective for example)?
- (final question) Is there anything else that you would like to add/share? Is there any related topic we haven’t mentioned that you would like to put fort?

*(Additional questions for non-farmers interviewees)*

- Do you see any levers or solutions to address the challenges you mentioned?
- In your opinion what are the farmers major motives for adopting BMPs?
- What do you think are the major hindrances or blockages that prevent farmers to adopt BMPs?

*(Additional questions for farmers interviewees)*

- Could you tell me what were your major motives to adopt certain of these BMPs?
- Could you tell me what were the main reasons for which you didn’t apply certain of these BMPs?
- Could you explicit on the reasons why you couldn’t implement those practices?
- Could you explicit on the reasons why you didn’t want to implement these practices?
