# Supplementary material for: Navigating agricultural nonpoint source pollution governance: A social network analysis of best management practices in central Pennsylvania
Source: PLoS One. 2024 May 23;19(5):e0303745. doi: 10.1371/journal.pone.0303745 (PMC11115221; doi:10.1371/journal.pone.0303745)
Supplement: S1 Table — (DOCX) [file pone.0303745.s004.docx]

**S2 Table**

**List and description of BMPs.**

| **ID** | **BMP** |  | **Description** |  | **Source** |
| --- | --- | --- | --- | --- | --- |
| **1** | **Forested riparian buffers** |  | Trees, shrubs and other plants that grow next to streams and rivers, also called forest buffers, or streamside forest buffers. |  | Chesapeake Bay Program: https://www.chesapeakebay.net |
| **2** | **Grass riparian buffers** |  | Grasses grown next to streams and rivers, also called forest buffers, or streamside forest buffers. |  | Chesapeake Bay Program: https://www.chesapeakebay.net |
| **3** | **Cover-crops (unharvested)** |  | Grown to fill in bare soil, provide soil cover and prevent erosion. Can be annual, biannual or perennial plants grown in a single or mixed stand during all or part of the year, including the non-growing season. |  | Chesapeake Bay Program: https://www.chesapeakebay.net |
| **4** | **No-till or conservation tillage** |  | Planting crops directly into prior crop residues or cover crop with minimal or no tillage. Conservation tillage leaves one-third or more of a farm field covered with crop residue or vegetation throughout the year. |  | Conservation District of Lancaster county: http://lancasterconservation.org/; and Chesapeake Bay Program: https://www.chesapeakebay.net |
| **5** | **Prescribed grazing** |  | Also called pasture management: managing grazing or browsing animals in order to improve the health and vigor of the pasture’s forage stand, while considering stocking rates, size of animals, resting periods, and seasonal changes. |  | Penn State Extension: https://extension.psu.edu/ |
| **6** | **Permanent grasslands or wetlands restoration/conservation** |  | Conservation = leaving a permanent and/or natural grassland unexploited for production; restoration is done on lands that were previously drained, |  | Penn State Extension: https://extension.psu.edu/ |
| **7** | **Contour grass strips or strip cropping** |  | Growing crops in contoured strips so that protective strips of grass, or close growing crops, are alternated with strips of row crops. |  | Conservation District of Lancaster county: http://lancasterconservation.org/; and Chesapeake Bay Program: https://www.chesapeakebay.net |
| **8** | **Hedgerows plantation/preservation** |  | Also called fencerows or strip habitats: a wildlife corridor, or habitat strip, proving food and cover for wildlife, sometimes connecting two habitats, such as two woodlots on a farm. |  | Penn State Extension: https://extension.psu.edu/ |
| **9** | **Precision feeding** |  | Managing the quantity and form of nitrogen and phosphorous fed to livestock and poultry to minimize the amount of those excreted in manure and the loss to the environment. It addresses how the ration is formulated and implemented on the farm, based on confirmed nutrient analyses of feed, forages, and by-products. |  | Penn State Extension: https://extension.psu.edu/ |
| **10** | **Sustainable stocking density** |  | Optimal/sustainable/ or reduced number of animal units, allowing the best utilization of the forage and animal performance while still maintaining a healthy forage stand (indicatively < 1.5 AUE / ha, or < 3.7 AUE / ac). |  | Penn State Extension: https://extension.psu.edu/ |
| **11** | **Animal exclusion (from waterways)** |  | Streambank crossing (stabilized area or structure constructed through a stream to provide a travel way for people, livestock, equipment, or vehicle), streamside fencing (fences along waterways to exclude livestock, can be woven wire or electric, and permanent or moveable. |  | Penn State Extension: https://extension.psu.edu/ |
| **12** | **Manure storage facility (, etc.)** |  | A concrete or lined earthen structure to temporarily store manure and barnyard runoff until crop conditions allow for spreading. Has to have sufficient storage capacity, be watertight, and above water table. |  | Penn State Extension: https://extension.psu.edu/ |
| **13** | **Having a manure management plan (written down)** |  | The fact that a farmer who applies (generated or imported) manure or agricultural process wastewater, has a written Manure Management Plan. If the farm is not a Concentrated Animal Feeding Operation (CAFO) Animal Concentration Area (ACA), Manure Management Plans can be prepared by the farmer. |  | Penn State Extension: https://extension.psu.edu/ |
| **14** | **Following a manure management plan** |  | The fact that a farmer who applies (generated or imported) manure or agricultural process wastewater, follows (implements) a Manure Management Plan. If the farm is not a Concentrated Animal Feeding Operation (CAFO) Animal Concentration Area (ACA), Manure Management Plans can be prepared by the farmer. CAFOs or CAOs are required to develop more detailed written plans, called Nutrient Management Plans. These plans must be developed by a Certified Nutrient Management Specialist. |  | Penn State Extension: https://extension.psu.edu/ |
| **15** | **Following a nutrients management plan (manure and fertilizers)** |  | Written, site-specific plan that reduces nutrient pollution while maintaining crop production. Contain a field’s crop production potential, the amount of nutrients needed to achieve this level of production and the recommended application amount, form, source, rate, placement and timing of manure or fertilizer. CAFOs and ACAs are required to developpe and follow Nutrient Management Plans, that have to be developed by a Certified Nutrient Management Specialist. |  | Chesapeake Bay Program: https://www.chesapeakebay.net |
| **16** | **Precision fertilization technology (and/or manure injection)** |  | Precision fertilization: variable-rate application of nutrients and pesticides and promote the use of GPS-enabled precision agricultural technology and equipment. Manure injection: injecting manure into the soil instead of spreading it on the surface. |  | USDA-NRCS: https://www.nrcs.usda.gov/; and Penn State: https://news.psu.edu/ |
