# Supplementary material for: Navigating agricultural nonpoint source pollution governance: A social network analysis of best management practices in central Pennsylvania
Source: PLoS One. 2024 May 23;19(5):e0303745. doi: 10.1371/journal.pone.0303745 (PMC11115221; doi:10.1371/journal.pone.0303745)
Supplement: S2 Table — (DOCX) [file pone.0303745.s005.docx]

**S4 Table**

**List and description of the organizations from the socio-centric network.**

| **Actor** |  | **Description** |
| --- | --- | --- |
| Farmers |  | Farmers, agricultural land managers, agricultural landowners |
| Conservation District |  | Conservation District (county level), under State Conservation Commission and Environmental Protection Agency |
| Chesapeake Bay Foundation |  | Private non-profit organization for the preservation of Chesapeake Bay, operating at watershed scale |
| PA-DCNR |  | State Department of Conservation and Natural Resources |
| NRCS local branch |  | Natural Resources Conservation Services agency of central Pennsylvania |
| ClearWater Conservancy |  | Central PA water quality conservation/restauration NGO |
| Universities |  | Public institutions of higher education (mainly Penn State, also Wisconsin, Maryland, and other universities from the region or neighboring regions) |
| No Till Alliance |  | Private non-profit farmers led organization focusing on soil conservation (Pennsylvania) |
| State Conservation Commission |  | Government agency under US-Department of Agriculture and US-Environmental Protection Agency |
| South-West Project Grass |  | Multi-actor partnership (farmers, Conservation Districts and agricultural industries) for the development of grazing practices |
| Xerces Society |  | Private non-profit organization for pollinators conservation |
| PA-DEP |  | Pennsylvania Department of Environmental Protection |
| PA-DCED |  | Pennsylvania Department of Community and Economic Development |
| PENNVEST |  | Public funding agency (grants and loans attribution) for infrastructure projects that have a positive impact on water quality |
| Northeast Cover Crops Council |  | Private non-profit organization for the development of cover crops |
| Private nature and service clubs |  | Local private nonprofit organizations for the preservation of natural habitat and nature recreation areas |
| USDA-ARS (PSWMRU) |  | US-Department of Agriculture - Agricultural Research Service (Pasture Systems & Watershed Management Research: University Park) |
| Larger public |  | Civil society |
| Agricultural industries |  | Agricultural businesses and industries (private for-profit) |
| Governor's Office |  | Governor's Office of the Commonwealth of Pennsylvania |
| PASA |  | Pennsylvania Association for Sustainable Agriculture (private non-profit organization, with a majority of farmers on the board) |
| SWCS |  | Soil and Water Conservation Society (private non-profit scientific and educational organization for natural resource conservation) |
| Local watershed associations |  | Local watershed associations from central Pennsylvania |
| Stroud Water Research Centre |  | Private non-profit organization for research, education and extension agency for soil and water conservation |
| NEMA Agroforestry |  | Northeast-Middle Atlantic Agroforestry: multi-actor partnership (network of researchers, technical service providers, agency staff, farmers) for the development of agroforestry |
| PAGLC |  | Pennsylvania Grazing Land Coalition (private non-profit for the development of grazing practices) |
| Rodale Institute |  | Private non-profit advocacy organization, research and extension for the promotion of organic agriculture |
| Dairy Grazing Apprenticeship |  | Private non-profit organization for beginning farmers (grazing ranchers) proving education and mentorship |
| Veterinarians |  | Private for-profit veterinarians |
| Animal nutritionists |  | Private for-profit livestock nutrition consultant |
| FSA local branch (central PA) |  | Farm Service Agency, administration of agricultural policies implementation in central Pennsylvania (under USDA) |
| USDA FSA (PA) |  | Farm Service Agency, state level |
| USDA (national) |  | US Department of Agriculture (federal level) |
| USDA-NAC |  | National Agroforestry Center (under USDA) |
| US-EPA (national) |  | United States Environmental Protection Agency (federal level) |
| William Penn Foundation |  | Foundation for the financial support of access to education, culture, public and natural spaces protection across PA's larger region |
| Chesapeake Conservation Partnership |  | Multi-actor partnership for the Chesapeake Watershed Implementation Plans (including the Agricultural Workgroup) |
| Chesapeake Bay Commission |  | Partnership of legislators for multistate coordination of Chesapeake Bay Watershed Programs (policy advise) |
| NRCS (PA) |  | Natural Resources Conservation Service, state level (under USDA) |
| Media |  | Social media, newspapers, magazines, radio, internet, including media specialized in agriculture or environment |
| Schools (primary, secondary) |  | Primary or secondary education schools |
| Private consultants |  | Agricultural consultants (private for-profits), individual or firms |
| Farmland Trust (Centre county) |  | Private non-profit organization for land trusts and conservation easements establishment in Centre county |
| PA Land Trust Alliance |  | Private non-profit for the facilitation of land trusts and conservation easements establishment across Pennsylvania (umbrella of several local land trusts) |
| Chesapeake Conservancy |  | Private non-profit organization for the conservation of Chesapeake Bay (larger watershed scale) |
| Farm Bureau (Centre county) |  | Trade association: local branch of farmers and agricultural industries association (private for profit, lobby) |
| NE Pasture Consortium |  | Multi-actor partnership for the development, conservation and sustainable use of pasture land |
| PA Farm Bureau |  | Umbrella of center level Farm Bureaus: trade and industry association, lobbying group |
| Pheasants Forever |  | Private non-profit wildlife and natural habitat conservation association |
| PennAg (industry association) |  | Private for-profit trade and agricultural industry association (lobby) |
| Farm Bureau (national) |  | Trade association: national branch of farmers and agricultural industries association (private for-profit, lobby) |
| Ag Land Preservation Board |  | Centre county committee for the preservation of agricultural land and conservation easements contraction |
| Local Government Boards |  | County, township, borough, or municipality administration boards |
| MSF4 |  | Municipal Separated Storm Sewer System: partnership of boroughs, townships of Spring Creek watershed |
| UAJA |  | University Area Joint Authority: multi-actor partnership for sewage system plant in part of Centre county |
| Pennfuture |  | Private non-profit organization for air, water, and land preservation |
| NE-SARE |  | Northeast Sustainable Agriculture Research and Education (part of USDA NIFA) |
