# Supplementary material for: Navigating agricultural nonpoint source pollution governance: A social network analysis of best management practices in central Pennsylvania
Source: PLoS One. 2024 May 23;19(5):e0303745. doi: 10.1371/journal.pone.0303745 (PMC11115221; doi:10.1371/journal.pone.0303745)
Supplement: S3 Table — (DOCX) [file pone.0303745.s006.docx]

**S6 Table**

**Codes and themes from interviews content analysis.**

| **BMPs/policies calibration, effectiveness, and adoption** | (un)effectiveness | (doubts on) BMPs or policy programs effectiveness |
| --- | --- | --- |
|  | (un)realistic | practical/technical (in)feasibility, (un)realism of the measures, taking into consideration farmers constrains (weather, investment, time, space, equipment, etc.) |
|  | climate | climate (change) influencing farmers decisions and practices |
|  | diversity and (mis)calibration | diversity/heterogeneity in farm types, management choices, socio-environmental contexts, and (mis)calibration of policy programs/measures to specific contexts |
|  | livestock distribution | (change in) spatial distribution and concentration of livestock at landscape/region or farm scale |
|  | selectivity | excessive, insufficient or intentional focus on certain BMPs, on certain farm types, or on certain criteria for regulation or attribution of support (financial, technical) |
| **Interests, (co)benefits and tradeoffs** | interests (conv./div.) | mutual/convergent/synergetic interests or to divergent interests/conflict of interests between actors |
|  | tradeoffs or co-benefits | antagonist tradeoffs or co-benefits/synergetic effects (environmental, economic or social) resulting from land-use choices |
| **Knowledge, exposure, connectedness** | (im)mobility | farmers geographic isolation versus propensity to mobility, to travel and see different places, contexts and systems |
|  | collaboration | multi-actor and multi-scale collaborations, coordination between actors of/for (different but interlinked) projects |
|  | education/knowledge and outreach/exposure | education, knowledge exchange and dissemination (including horizontal transfer and mutual exchange), means of communication and outreach, connectedness, exposure and receptivity networks and different actors |
| **Mindset** | (in)dependence | relying on other actors, programs or policy measures versus will to be/remain independent, not to be tied to other agencies or organizations |
|  | innovation, adaptability/flexibility | will/capacity or aversion to innovate, adapt to change and implement change |
|  | priorities | BMPs (not) considered as a priority to direct time, energy and money resources in |
|  | risk | risk (aversion, mitigation and prevention), including economic, climatic, technical failures risks |
|  | tradition, culture | mindset/behavior linked to habits, identity, tradition, culture, family and peer pressure, including social risk (fear of judgement, of loss of acceptance and recognition) |
| **Actors and policies (roles and perceptions)** | farmers | role of, relation to and representation of (peer) farmers and the farming community |
|  | government | role of, relation to and representation of government (agencies) |
|  | private for-profit | role of, relation to and representation of the private for-profit/corporate sector |
|  | private non-profit | role of, relation to and representation of non-profits and non-governmental advocacy groups |
|  | regulations and enforcement | (un)acceptance, (positive or negative) effect(s), and perception of enforcement, regulations and mandatory policy measures |
|  | voluntary measures | (un)acceptance, (positive or negative) effect(s), and perception of enforcement voluntary policy measures/programs |
| **Resources allocation and economic valuation** | cost-share | farmers (in)capacity to cover BMPs cost-share, (un)ability to invest to set-up BMPs and cover their maintenance costs |
|  | funding | lack of, need for, or availability of funding, revenue sources to finance BMPs implementation and maintenance |
|  | human and time resources | manpower and/or time *(un)availability / lack of, need for, or availability of technical staff |
|  | valuation | (economic) valuation of environmental and social outputs/services provided by farmers and land managers |
|  | viability | farms economic (un)viability, farmers (in)capacity to generate (sufficient, decent) income |
| **Social responsibility and recognition** | inclusion | inclusion/integration (or not) of land managers in discussion, decision and design of policies, making use (or not) of local knowledge |
|  | power | distribution and concentration of power, degree of and exertion of control and influence |
|  | public behavior and perception | consumers' and the general public's behavior and perception of actors (farmers, government, NGOs, companies, etc.) and policies |
|  | recognition | social recognition and upliftment of land stewardship, of farmers skills, of farmers contribution to society and to public good |
|  | representation | feeling of (not) being represented, of (not) having a voice |
|  | responsibility distribution | (un)equitable distribution of responsibilities, blame/pressure, roles/expectations |
|  | stewardship | stewardship, care of the land, taking ownership and responsibility of the land |
